# Supplementary material for: Galangal–Cinnamon Spice Mixture Blocks the Coronavirus Infection Pathway through Inhibition of SARS-CoV-2 MPro, Three HCoV-229E Targets; Quantum-Chemical Calculations Support In Vitro Evaluation
Source: Pharmaceuticals (Basel). 2023 Sep 28;16(10):1378. doi: 10.3390/ph16101378 (PMC10610207; doi:10.3390/ph16101378)
Supplement: Supplementary file 1 [file pharmaceuticals-16-01378-s001.zip › pharmaceuticals-2506144-supplementary.pdf]

Cpd.	E_{Lumo}	E_{Homo}	ESP surface
**Rutin**	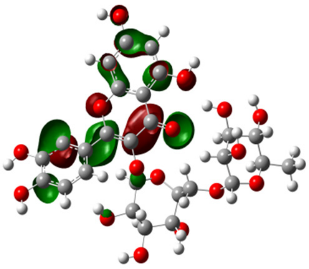	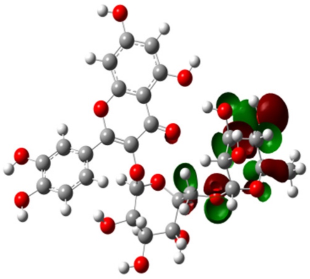	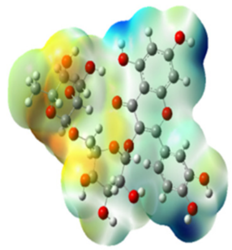
**Catechin**	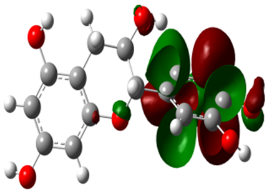	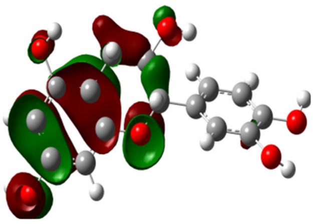	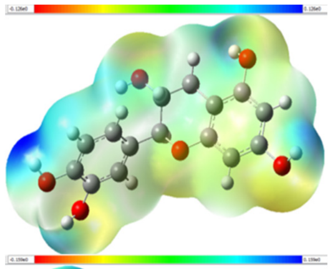
**Quercetin**	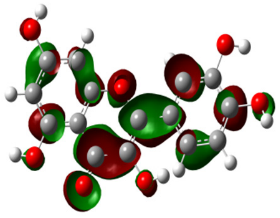	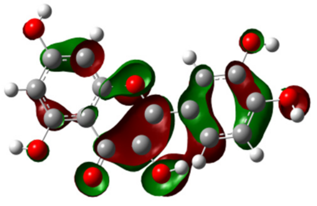	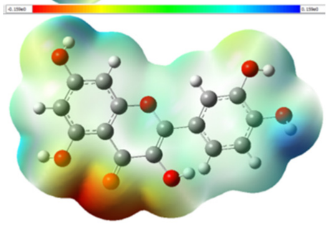
**Ellagic acid**	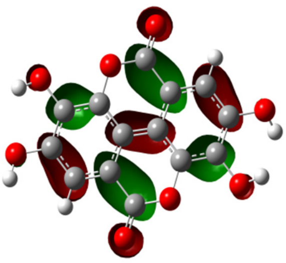	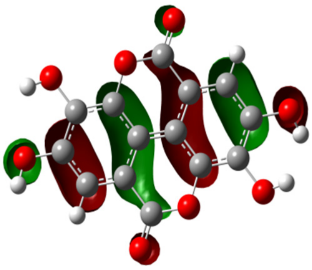	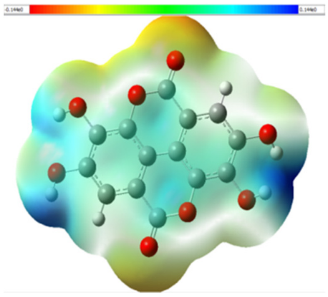

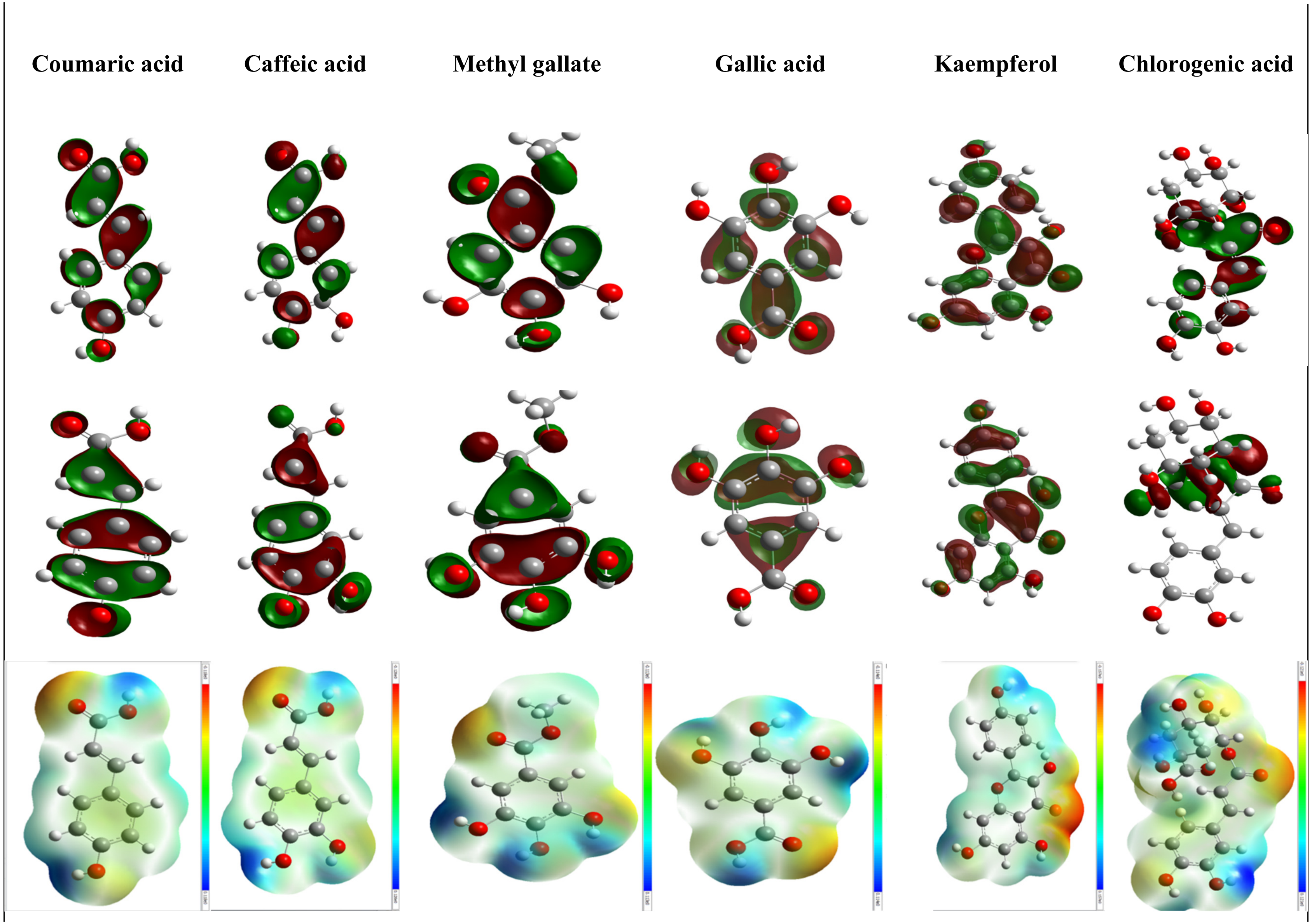
Coumaric acid
Caffeic acid
Methyl gallate
Gallic acid
Kaempferol
Chlorogenic acid

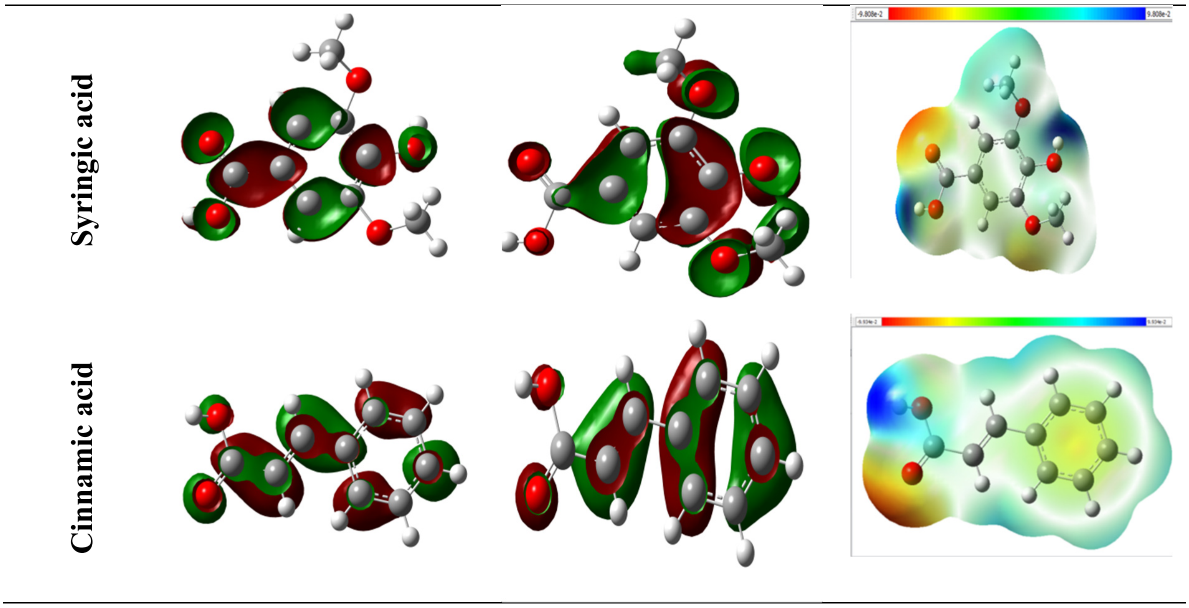

Figure S1. Frontier molecular orbitals (HOMOs, LUMOs) and Molecular electrostatic potential (MEP) surface of all detected phenolic compounds by using DFT/B3LYP/6-311++G(2d,2p) method

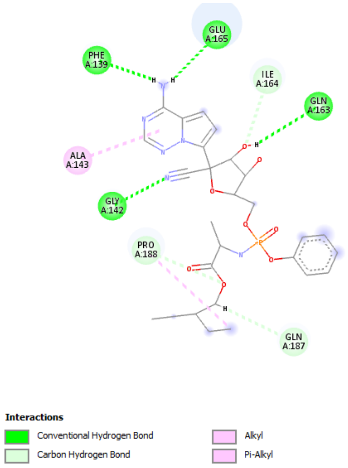

Remdesivir

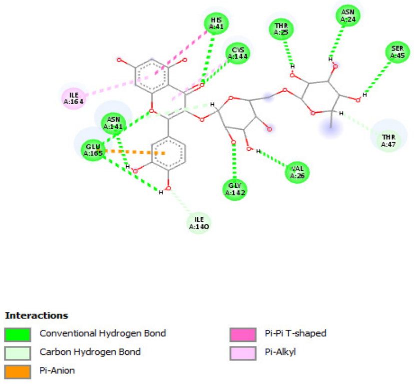

Rutin

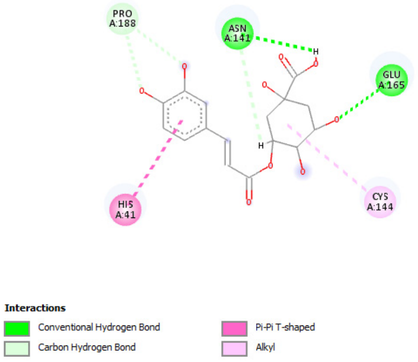

Chlorogenic acid

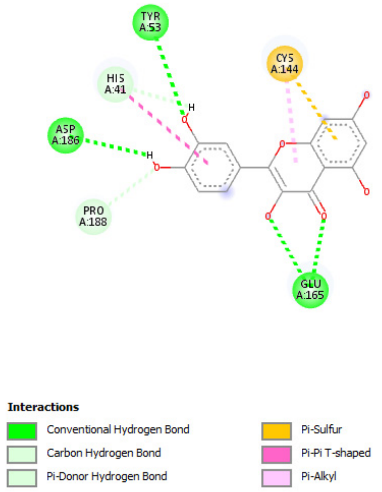

Quercetin

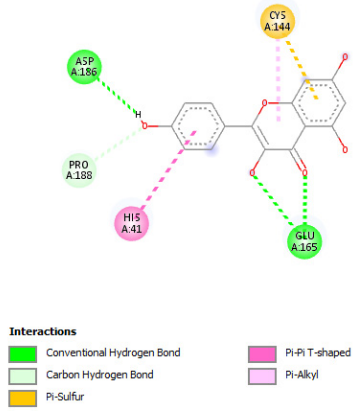

Kaempferol

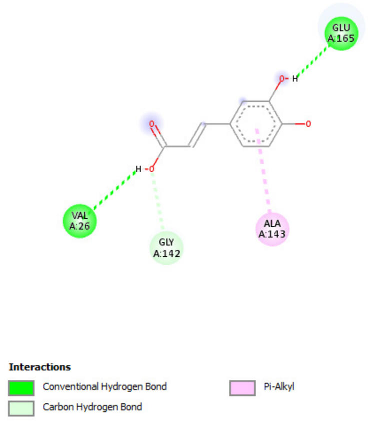

Caffeic acid

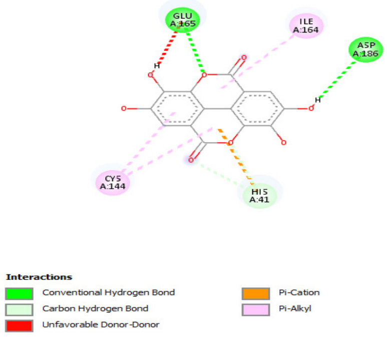

Ellagic acid

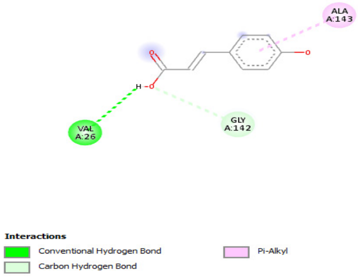

Coumaric acid

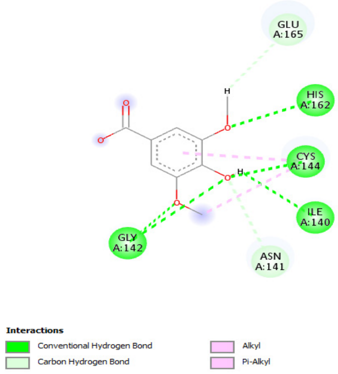

Syringic acid

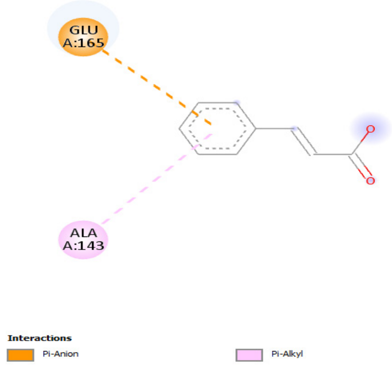

Cinnamic acid

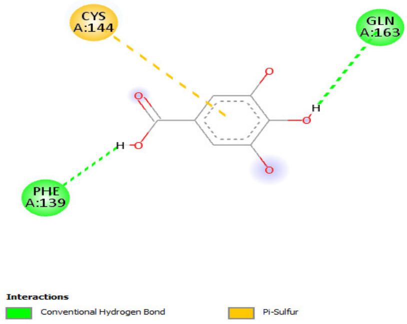

Gallic acid

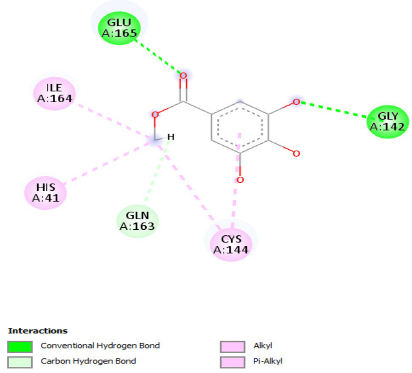

Methyl gallate

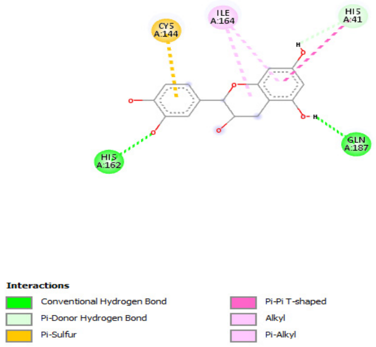

Catechin

Figure S2. 2D interactions 12 phenolic compounds of GCAE and Remdesivir docked on HCoV-229E M^{pro} (PDB ID: 2ZU2)

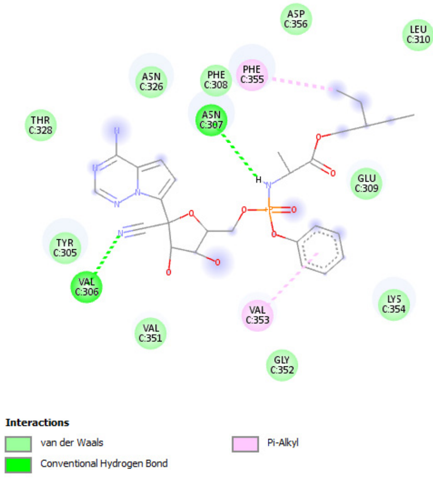

Remdesivir

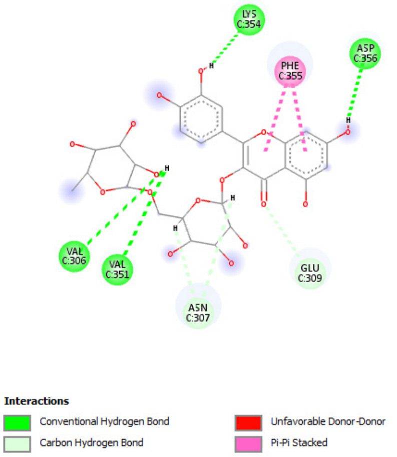

Rutin

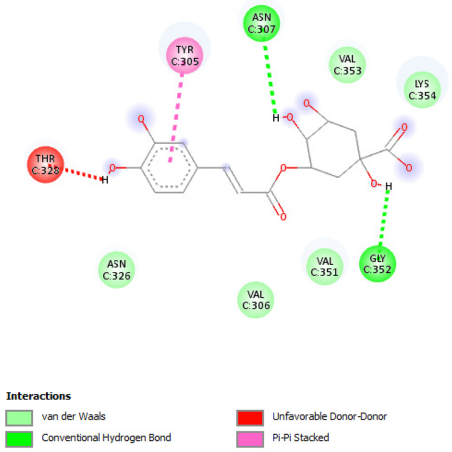

Chlorogenic acid

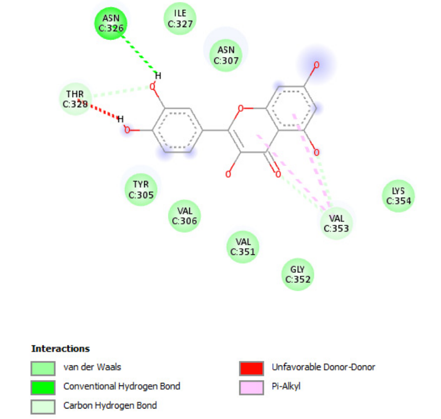

Quercetin

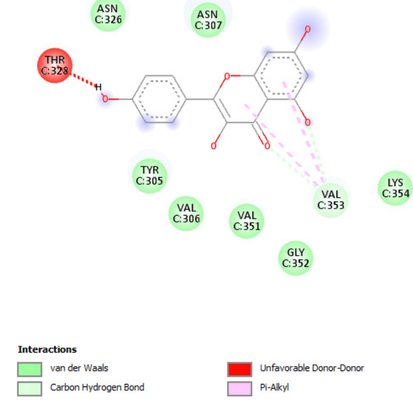

Kaempferol

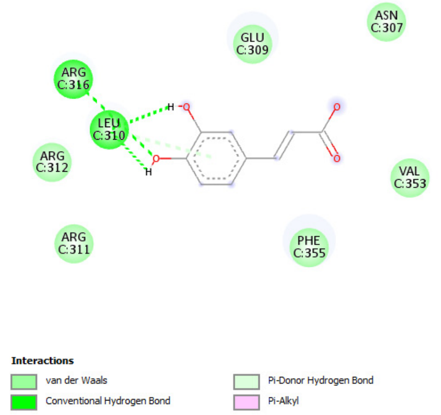

Caffeic acid

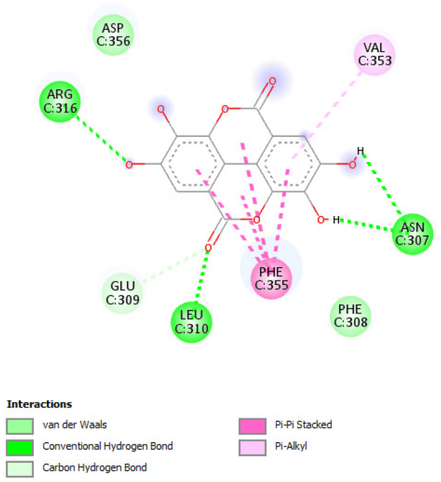

Ellagic acid

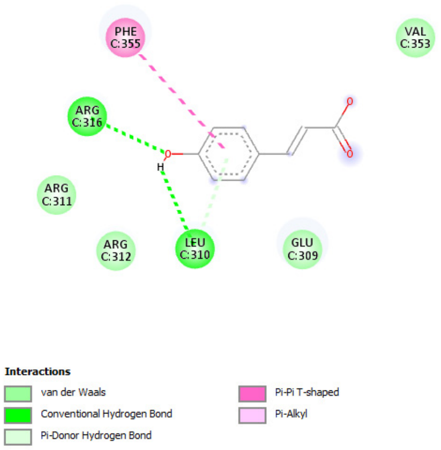

Coumaric acid

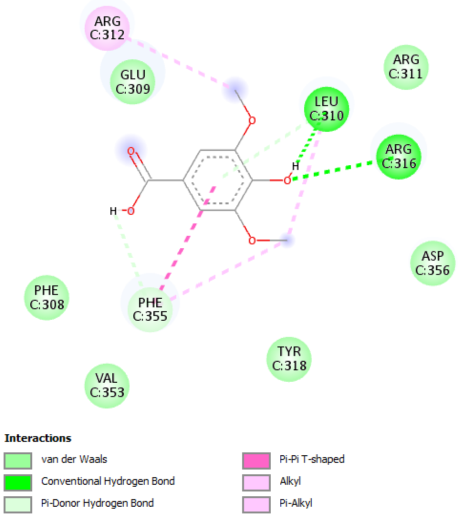

Syringic acid

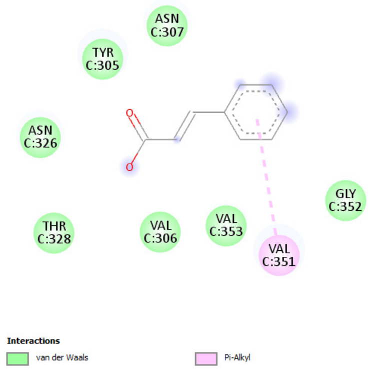

Cinnamic acid

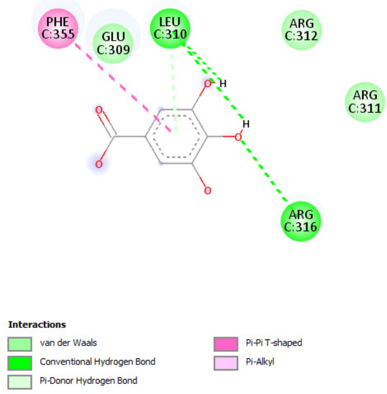

Gallic acid

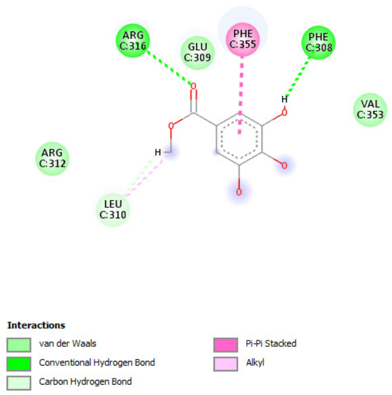

Methyl gallate

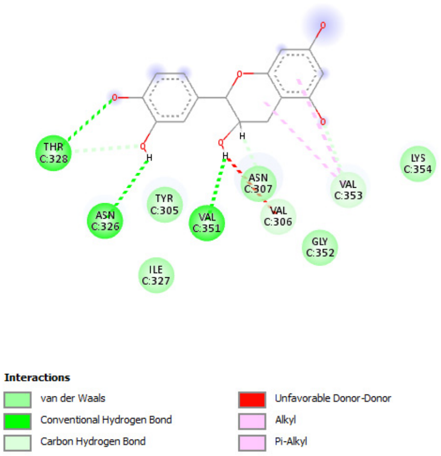

Catechin

Figure S3. 2D interactions 12 phenolic compounds of GCAE and Remdesivir docked on HCoV-229E RBD Class V (PDB ID: 6U7G)

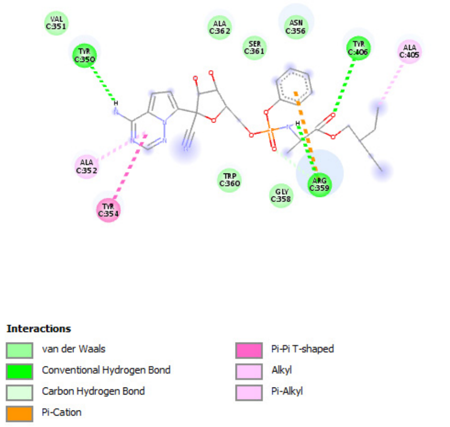

Remdesivir

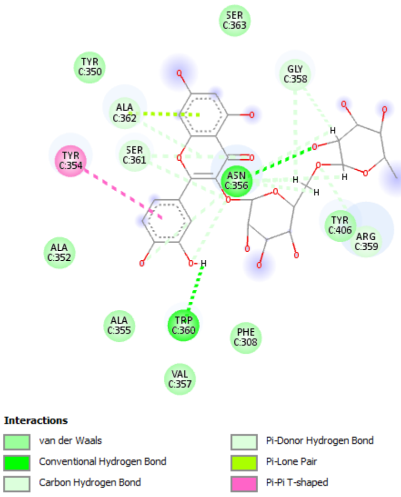

Rutin

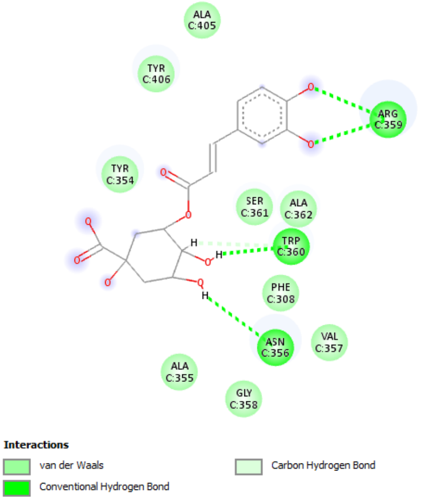

Chlorogenic acid

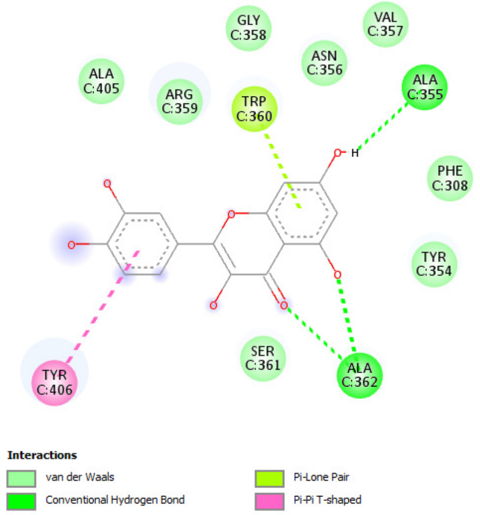

Quercetin

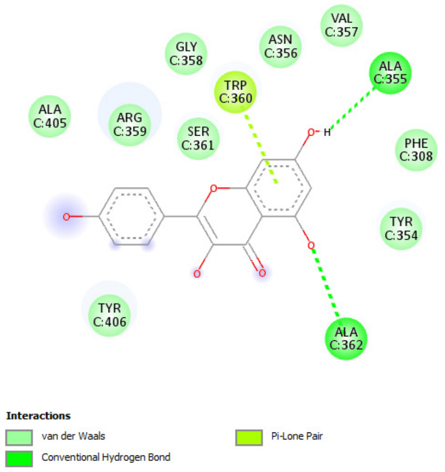

Kaempferol

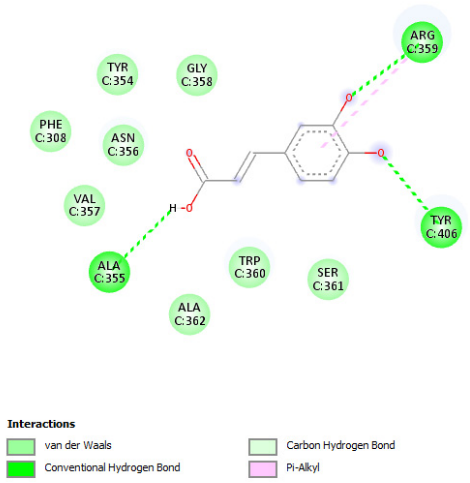

Caffeic acid

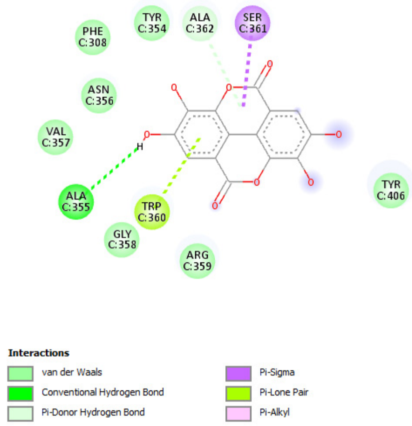

Ellagic acid

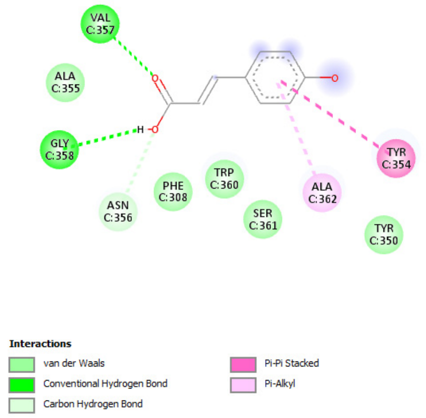

Coumaric acid

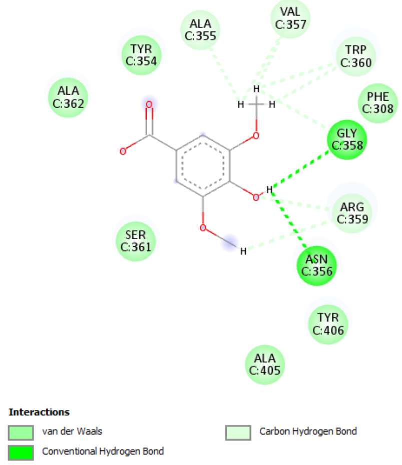

Syringic acid

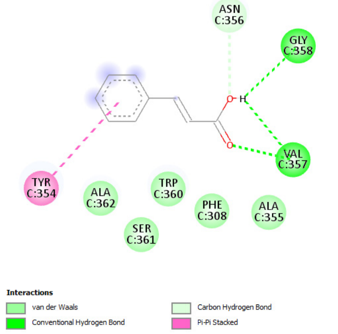

Cinnamic acid

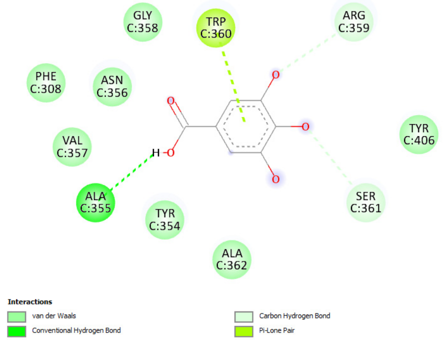

Gallic acid

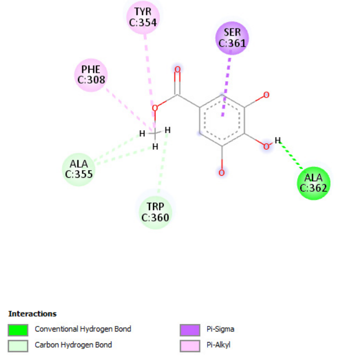

Methyl gallate

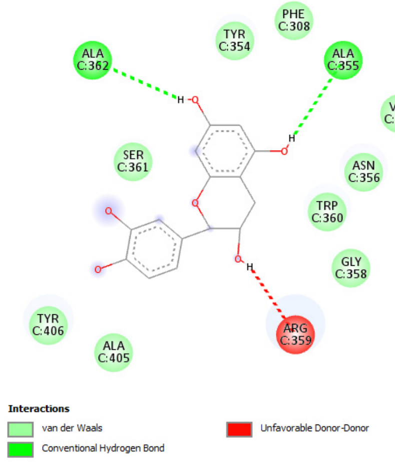

Catechin

Figure S4. 2D interactions 12 phenolic compounds of GCAE and Remdesivir docked on HCoV-229E spike protein (PDB ID: 7VN9)

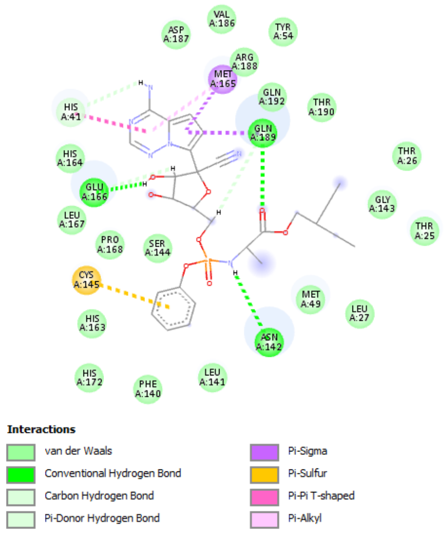

Remdesivir

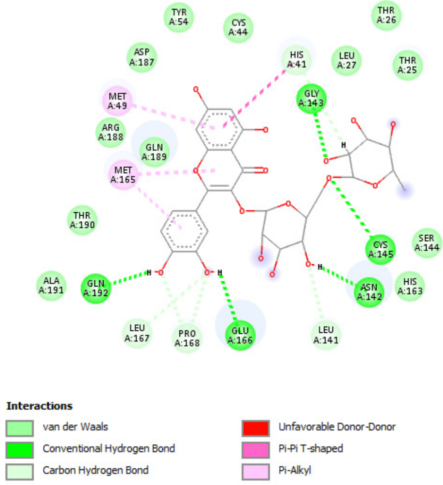

Rutin

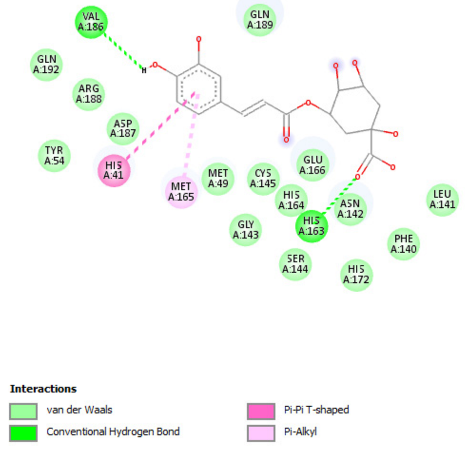

Chlorogenic acid

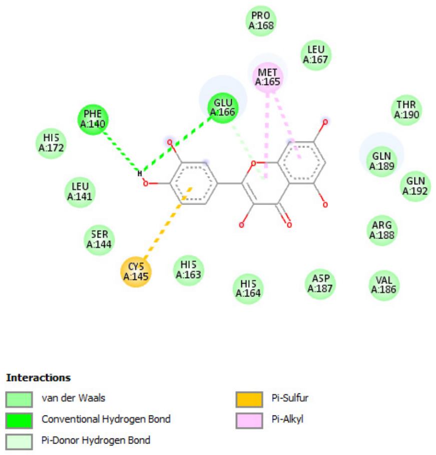

Quercetin

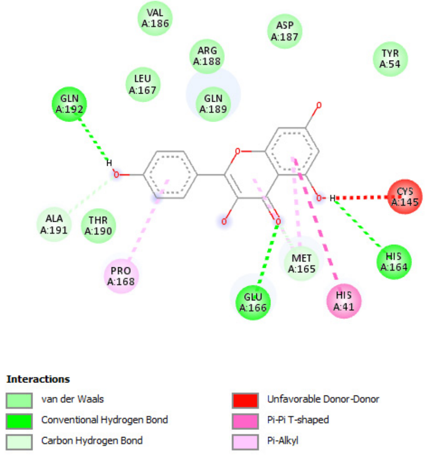

Kaempferol

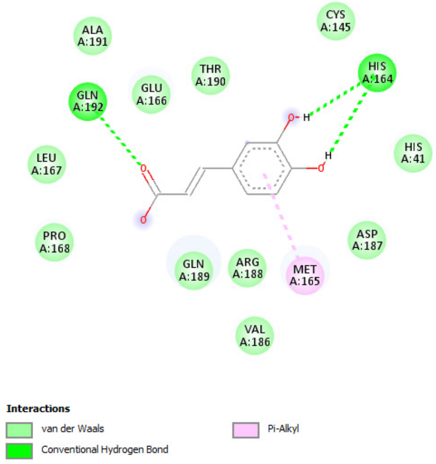

Caffeic acid

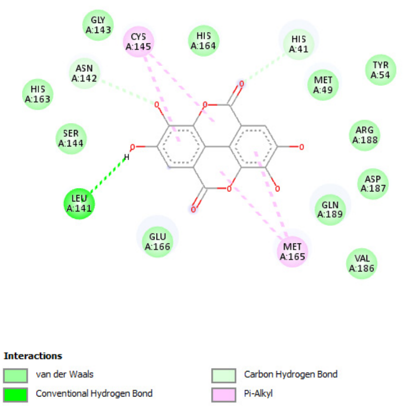

Ellagic acid

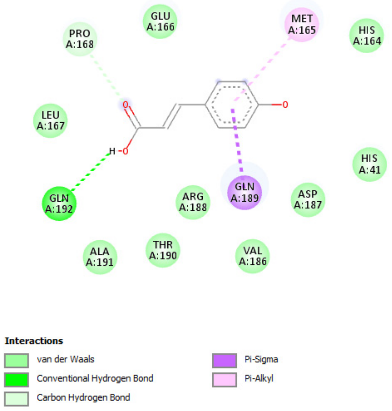

Coumaric acid

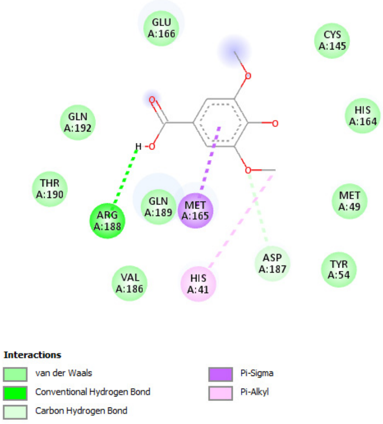

Syringic acid

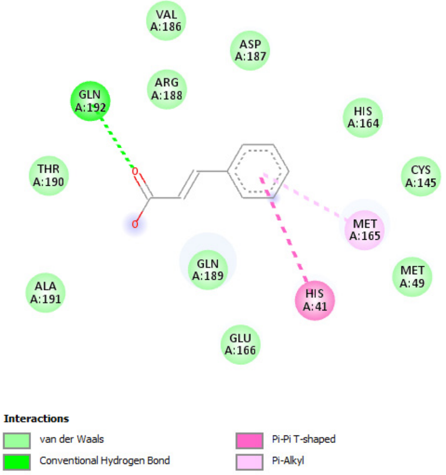

Cinnamic acid

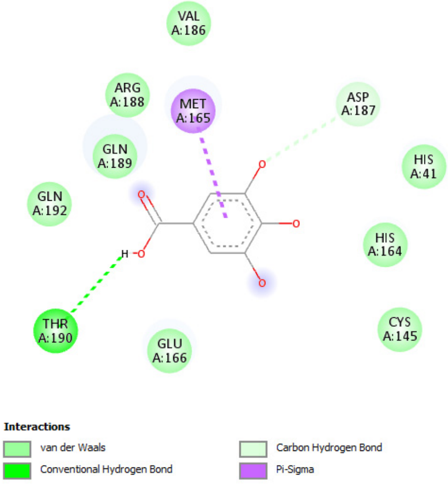

Gallic acid

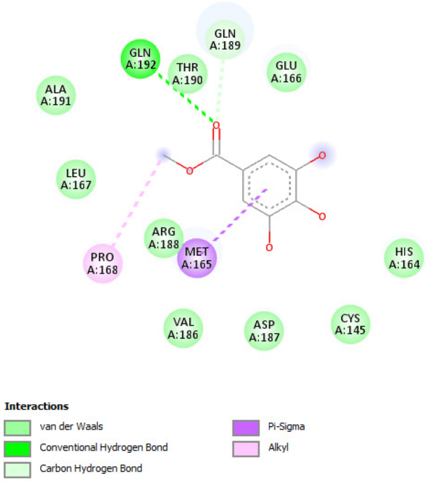

Methyl gallate

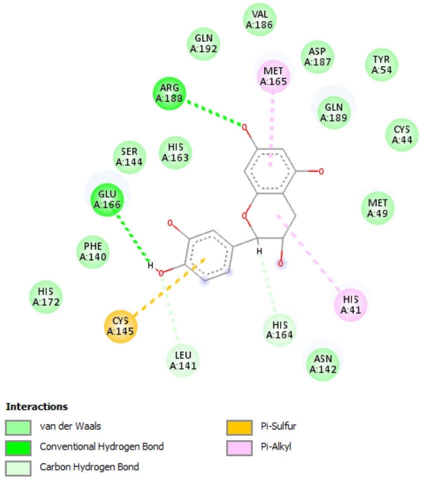

Catechin

Figure S5. 2D interactions 12 phenolic compounds of GCAE and Remdesivir docked on SARS-CoV-2 M^{pro} (PDB ID: 6WTT)
